# Platelet-rich plasma (PRP) augmentation does not result in more favourable outcomes in arthroscopic meniscal repair: a meta-analysis

**DOI:** 10.1186/s10195-022-00630-1

**Published:** 2022-02-07

**Authors:** Filippo Migliorini, Francesco Cuozzo, Lucio Cipollaro, Francesco Oliva, Frank Hildebrand, Nicola Maffulli

**Affiliations:** 1 Department of Orthopaedic, Trauma, and Reconstructive Surgery, RWTH University Hospital, Pauwelsstraße 30, 52074 Aachen, Germany; 2 Department of Medicine, Surgery and Dentistry, University of Salerno, 84081 Baronissi, SA Italy; 3 Clinica Ortopedica, Ospedale San Giovanni di Dio e Ruggi d’Aragona, 84131 Salerno, Italy; 4 School of Pharmacy and Bioengineering, Faculty of Medicine, Keele University, Thornburrow Drive, Stoke on Trent, England; 5 Queen Mary University of London, Barts and the London School of Medicine and Dentistry, Centre for Sports and Exercise Medicine, Mile End Hospital, 275 Bancroft Road, London, E1 4DG England

**Keywords:** Meniscus, Repair, Arthroscopy, Augmentation, PRP

## Abstract

**Background:**

The efficacy and safety of platelet-rich plasma (PRP) augmentation for arthroscopic meniscal repair is controversial. This meta-analysis compared arthroscopic meniscal repair performed in isolation or augmented with PRP.

**Methods:**

The present study was conducted according to PRISMA 2020 guidelines. Pubmed, Web of Science, Google Scholar and Embase were accessed in August 2021. All the clinical trials which compared arthroscopic meniscal repair performed in isolation or augmented with PRP were included.

**Results:**

Eight hundred thirty-seven patients were included: 38% (318 of 837 patients) were women; the mean age of the patients was 35.6 (range, 20.8–64.3) years; the mean follow-up was 26.2 (range, 6–54) months. Similarity was found in analogue scale (VAS) (*P* = 0.5) and Lysholm (*P* = 0.9), and International Knee Documentation Committee (IKDC) scores (*P* = 0.9). Similarity was found in the rate of failure (*P* = 0.4) and rate of revision (*P* = 0.07).

**Conclusion:**

The current published scientific evidence does not support PRP augmentation for arthroscopic meniscal repair.

## Introduction

Meniscal lesions are common, with an estimated prevalence of 12% in the adult population [1]. Arthroscopic meniscectomy has been widely performed to reduce pain deriving from these lesions and restore patients’ quality of life [2, 3]. Observational studies have demonstrated that meniscectomy is associated with early onset osteoarthritis [4–9]. Therefore, resection of the meniscal structures should be minimised or even avoided [2, 10, 11]. Patients with a meniscal tear but otherwise healthy meniscal tissue who wish to remain active may benefit from a meniscal repair [9, 12, 13]. Intra-meniscal injections of growth factors, including those present in platelets, could stimulate cell activity and favour meniscal healing [14–16]. The regenerative potential of platelet rich plasma (PRP) has been documented [15–18]. PRP is obtained by centrifugation of platelets extracted from peripheral venous blood [19, 20]. Given its regenerative properties (e.g. neoangiogenesis, proteins synthesis, cell proliferation and migration), PRP has been used in the conservative management of several knee ailments including osteoarthritis [21, 22] and meniscal lesions [21, 23, 24]. The efficacy of PRP augmentation has also been investigated in arthroscopic meniscal repair [15, 20, 25–31]. However, the results from these studies are controversial, and the actual benefit of PRP augmentation for arthroscopic meniscal repair is unclear. Therefore, a meta-analysis was conducted hypothesising that PRP augmentation in combination with arthroscopic meniscal repair would lead to greater patient-reported outcome measures (PROMs) and accelerate the healing process.

## Material and methods

### Eligibility criteria

All the clinical trials comparing arthroscopic isolated meniscal repair with meniscal repair augmented with PRP were accessed. According to the authors’ language capabilities, articles in English, German, Italian, French and Spanish were eligible. Only studies with evidence levels I–III, according to the Oxford Centre of Evidence-Based Medicine [32], were considered. Reviews, technical notes, comments, letters, editorials, protocols and guidelines were excluded. Biomechanical, computational, in vitro, animal and cadaveric studies were also not eligible. Only studies published in peer reviewed journals were eligible. Studies combining PRP with other procedures were not considered, nor were those augmenting arthroscopic meniscal repair with other compounds. Only studies reporting data with a minimum of 6 months follow-up were eligible. Studies evaluating experimental rehabilitation programs were not considered. Studies which performed mini-arthrotomy and/or those concerning meniscal repair in revision settings were not eligible. Only studies reporting quantitative data under the outcomes of interest were considered for inclusion.

### Search strategy

This systematic review was conducted according to the Preferred Reporting Items for Systematic Reviews and Meta-Analyses (PRISMA) guidelines [33]. The PICOT framework was followed:

- P (Population): meniscal tears;
- I (Intervention): isolated arthroscopic meniscal repair;
- C (Comparison): arthroscopic meniscal repair augmented with PRP;
- O (Outcomes): PROMs, complications;
- T (Timing): minimum 6 months follow-up.

In August 2021, the following databases were accessed: Pubmed, Web of Science, Google Scholar and Embase. No time constraints were used for the search. The following keywords were used in combination: *meniscal, menisci, augmentation, PRP, repair, combined, isolated, knee, arthroscopy, platelet-rich plasma, meniscopathy, damage, injury, tear, patient reported outcome measures, PROMs, Lysholm, IKDC, failure, complications, pain, revision, visual analogue scale*.

### Selection and data collection

Two authors (F.M.;F.C.) independently performed the database search. All the resulting titles were screened and, if suitable, the abstracts were accessed. The full-text of the abstracts which matched the topic were subsequently accessed. A cross reference of the bibliography of the full-text articles was also accomplished to identify additional articles. Disagreements were debated and solved by a third author (N.M.*).

### Data items

Two authors (**;**) independently performed data extraction. Study generalities (author and year, journal, study design, length of the follow-up) were collected. Patient demographic at baseline was retrieved: number of procedures, mean age, percentage of women, visual analogue scale (VAS), and time elapsed between the injury and arthroscopy. The following data were extracted at last follow-up: International Knee Documentation Committee (IKDC) [34], Lysholm score [35], VAS, rates of failure and revision. Failure was defined as the recurrence of meniscal symptoms or the request by the patient to repeat arthroscopy [20, 25].

### Study risk of bias assessment

The risk of bias was assessed using Review Manager 5.3 software (The Nordic Cochrane Collaboration, Copenhagen). The risk of bias was evaluated based on the guidelines in the Cochrane Handbook for Systematic Reviews of Interventions [36]. Two reviewers (**;**) evaluated the risk of bias of the extracted studies. The following endpoints were evaluated: selection, detection, performance, attrition, reporting and other bias. To assess the overall risk of publication bias, the funnel plot of the most commonly reported outcome was performed. The funnel plot charted the standard error (SE) of the log odds ratio (Log _OR_) versus its OR. The degree of asymmetry of the plot is directly proportional to the degree of bias. To assess the risk of bias of each included studies, a risk of bias graph was created.

### Synthesis methods

The statistical analyses were performed by the main author (**) using Review Manager 5.3 software (The Nordic Cochrane Collaboration, Copenhagen). For descriptive statistics, mean difference and standard deviation were used. A *t*-test was performed to assess baseline comparability, with values of *P* > 0.1 considered satisfactory. For continuous data, the inverse variance method with mean difference (MD) effect measure was used. For binary data, the Mantel–Haenszel method with odds ratio (OR) effect measure was used. The confidence interval (CI) was set at 0.95 in all the comparisons. Heterogeneity was assessed using $\chi$^2^ and Higgins-I^2^ tests. If $\chi$^2^ > 0.05, no statistically significant heterogeneity was found. If $\chi$^2^ < 0.05 and Higgins-I^2^ > 60%, high heterogeneity was found. A fixed model effect was used as default. In case of high heterogeneity, a random model was used. Overall values of *P* < 0.05 were considered statistically significant.

## Results

### Study selection

**Fig. 1:**
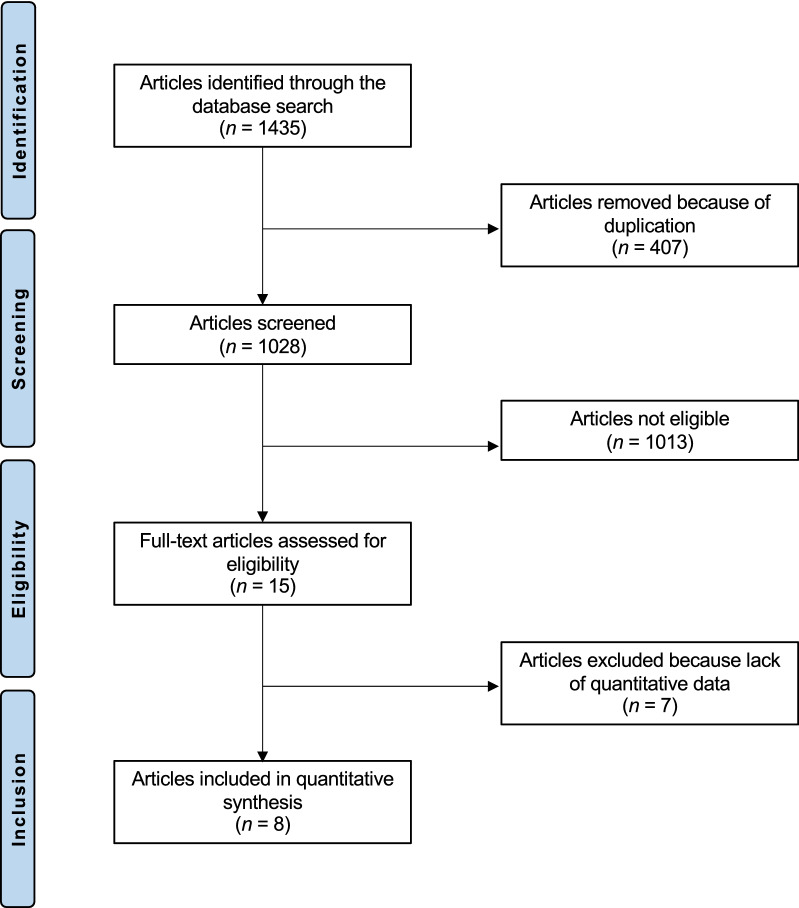
Flow chart of the literature search

The literature search resulted in 1435 articles. Of these, 407 articles were duplicates. A further 1013 articles were not eligible as they did not match the following inclusion criteria: study design (*N* = 298), non-comparative studies (*N* = 109), not matching the topic of the study (*N* = 582), combining multiple or experimental procedures (*N* = 13), short follow-up and/or limited study size (*N* = 3) and uncertain data (*N* = 8). A further seven studies were excluded as they did not report quantitative data under the outcomes of interest. Finally, eight studies were included for formal analysis. The literature search results are shown in Fig. 1.

### Methodological quality assessment

**Fig. 2:**
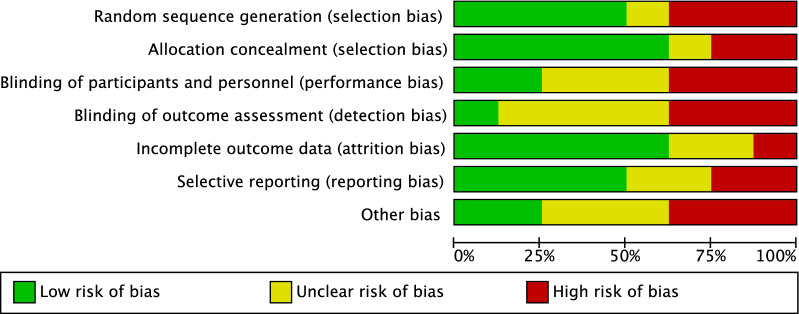
Methodological quality assessment

The limited number of randomized clinical trials (three out of nine studies) increased the risk of selection bias, which was low to moderate. The selection criteria were often biased, and the general heath measures were rarely reported. Given the lack of blinding in most studies, the risk of detection bias was moderate. Attrition and reporting biases were both low to moderate. The risk of other potential biases was moderate. In conclusion, the overall risk of bias among the included studies was moderate (Fig. 2).

### Risk of publication bias

**Fig. 3:**
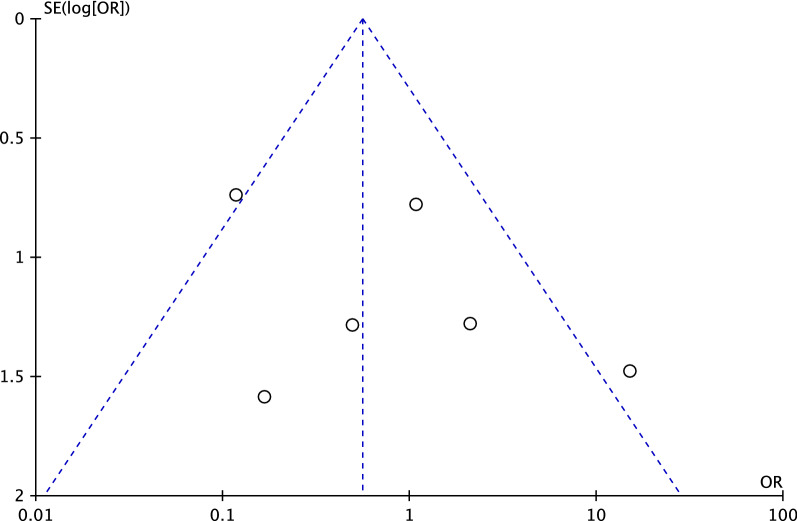
Funnel plot of the endpoint “revision”

To evaluate the risk of publication bias, the funnel plot of the most commonly reported outcome (rate of revision) was performed. The plot evidenced good symmetry, with most of the referral points included within the pyramidal shapes. In conclusion, the risk of publications bias was low (Fig. 3).

### Study characteristics and patient demographic

A total of 837 patients were included: 38% (318 of 837 patients) were women; the mean age of the patients was 35.6 (range, 20.8–64.3) years; the mean follow-up was 26.2 (range, 6–54) months. Good comparability was found between the two groups in terms of mean age, percentage of women, time elapsed between injury and arthroscopy, and VAS (*P* > 0.1). Concerning the centrifugation procedure, a median rate of 1500 rpm for the first centrifugation, followed by a second centrifugation at 3400 rpm was found, with a mean extracted venepuncture volume of 94.8 ml. The mean platelet concentration after preparation was significantly greater than that of blood. The demographics of the included studies are presented in Table 1.

**Table 1:** Generalities and patient baseline data of the included studies

Author, year	Journal	Design	Follow-up (*months*)	Treatment	Menisci (*n*)	Mean age	Female (%)
Dai et al. 2019, [25]	*BMC Musculoskel Disorder*	Retrospective	20.7	PRP	14	32.4	57
				No PRP	15	30.3	67
Duif et al. 2015, [26]	*Arch Orthop Trauma Surg*	Prospective, randomised	12	PRP	24	64.1	42
				No PRP	34	64.3	65
Everhart et al. 2019, [27]	*Am J Sport Med*	Prospective	36	PRP	203	30.0	36
				No PRP	347	28.1	37
Griffin et al. 2015, [28]	*Clin Orthop Relat Res*	Retrospective	24	PRP	15	26.0	27
				No PRP	20	35.0	15
Kaminski et al. 2018, [30]	*Biomed Res Int*	Prospective, randomised	54	PRP	21	30.0	21
				No PRP	18	26.0	17
Kaminski et al. 2019, [31]	*Int J Mol Sci*	Prospective, randomised	23	PRP	42	44.0	48
				No PRP	30	46.0	37
Kemmochi et al. 2018, [20]	*J Orth*	Prospective	6	PRP	17	32.4	47
				No PRP	5	20.8	40
Pujol et al. 2015, [15]	*Knee Surg Sports Traumatol Arthrosc*	Prospective	34	PRP	17	32.3	35
				No PRP	17	28.3	24

### Synthesis of results

**Fig. 4:**
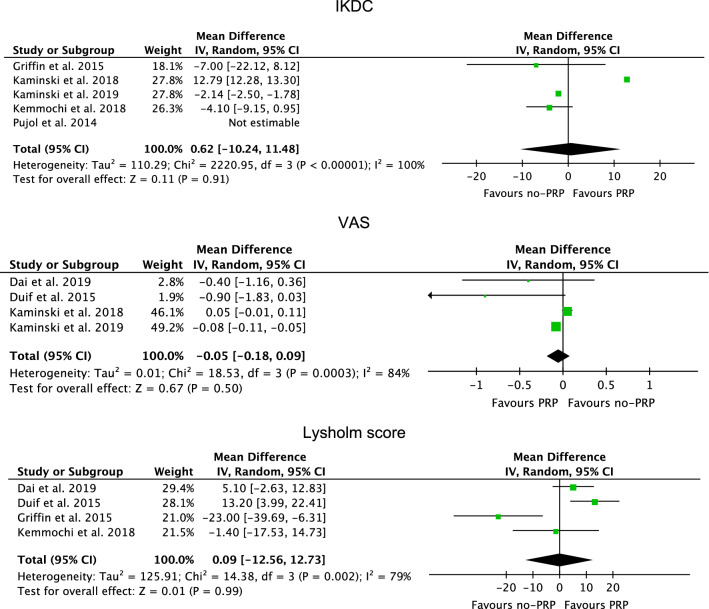
Forest plots of the comparisons: PROMs

Similarity was found in VAS (*P* = 0.5), Lysholm score (*P* = 0.99) and IKDC (*P* = 0.9) (Fig. 4).

**Fig. 5:**
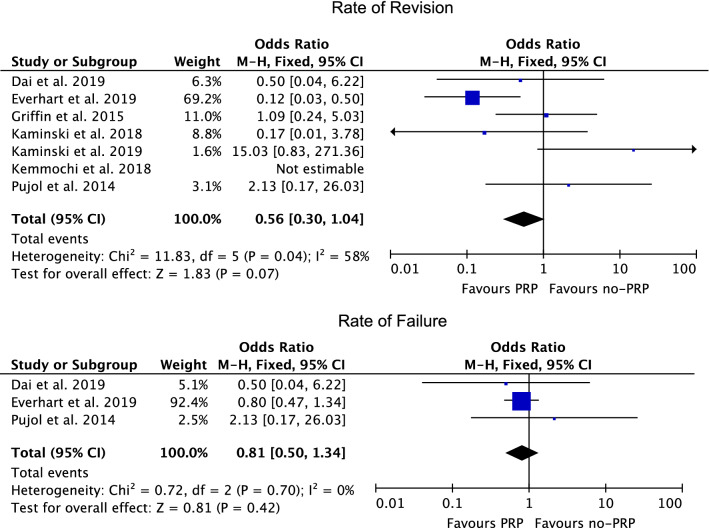
Forest plots of the comparisons: rate of complications

Similarity was found the rate of failure (*P* = 0.4) and rate of revision (*P* = 0.07) (Fig. 5).

## Discussion

According to the main findings of the present meta-analysis, the current level I of evidence does not support PRP augmentation for arthroscopic meniscal repair. At approximately 2 years follow-up, PRP augmentation demonstrated similar VAS, Lysholm, and IKDC scores compared with isolated arthroscopic meniscal repair. Moreover, no differences were detected in failure and revision rates. Following its introduction in the 1950s, PRP application has been employed for regenerative medicine purposes, and extended to musculoskeletal disorders since the 1980s [19, 37, 38]. Given their limited vascularisation and metabolic activity, the menisci of the knee exhibit poor regenerative capacity [39–41]. Growth factor injections may potentially activate the meniscal cells and stimulate regeneration [42, 43]. Platelets exhibit a high concentration of growth factors and mediators, such as transforming growth factor (TGF)-β, vascular endothelial growth factor (VEGF), epidermal growth factor (EGF), insulin-like growth factor 1 (IGF-1) and basic fibroblast growth factor (b-FGF), which enhance chemotaxis, angiogenesis, cell proliferation and extracellular matrix formation [42]. Therefore, injections of PRP are believed to accelerate healing and improve regeneration [43–48]. The benefits of PRP for cartilage regeneration have been recognised [49, 69, 70]. PRP reduces catabolism and increases the anabolic activity of hyaline cartilage [23]. Meniscal catabolic activity is crucial for osteoarthritis progression in the knee [50–52]. In vitro studies demonstrated the antinociceptive and proliferative properties of PRP, which increased extracellular matrix production and meniscal tissue regeneration [50, 53]. Furthermore, PRP improved meniscal cell viability in animal studies, increasing meniscal compressive strength through the overexpression of proteoglycans [54–56]. The current evidence of PRP augmentation for arthroscopic meniscal repair is controversial. Four of the included studies [25, 26, 30, 31] reported no statistically significant differences in pain assessment using VAS scores. Current evidence on the effects of PRP on pain is controversial [29, 56–58]. In the present investigation, 63% (five out of eight) studies reported no significant differences in IKDC scores in the PRP-augmented groups. The IKDC score was also compared in recent systematic reviews, with similar results [29, 59].

A recent systematic review evaluating six studies (309 patients) reached similar conclusions [29]. However, other investigations, evidenced controversial results. In a systematic review including five studies (82 procedures), PRP enhanced meniscal repair and resulted in a lower failure rate, but the evidence was not compelling enough to support the use of PRP in meniscal repair [57]. Another systematic review of five studies (274 procedures) concluded that PRP augmentation during arthroscopic meniscal repair is related to better outcomes and leads to significantly lower failure rates (from 26.7% to 50%) [59]. Similar findings were evidenced in a meta-analysis of six studies (111 procedures), in which PRP augmentation did reduce the risk of failure (from 25.7% to 9.9%) [60]. In 5323 procedures (83 studies), PRP resulted in better outcomes following meniscectomy [61]. Wang et al. [58], in a meta-analysis of 293 patients (six studies), found that PRP injection can improve the efficacy of arthroscopic meniscal repair, reducing the failure rate and severity of pain. This diversity may be explained by the heterogeneous criteria, dosage and procedures included, which led to variable results.

This meta-analysis has several limitations. The limited number of studies included and the relatively small size in the cohorts in the various investigations do not allow reliable conclusions to be inferred. The retrospective design of most of the included studies represents another important limitation. Moreover, between-study heterogeneity with regard to the length of follow-up was evident. Post-operative rehabilitation was seldom described, and the length of the follow-up was limited in most of the included studies. The description of surgical technique was not adequately reported in some studies, which represents a further limitation. Heterogeneity in PRP preparation and processing protocols was evident, as were between-study differences with regard to the initial whole blood volume and centrifugation rate and duration [62–65]. Battaglia et al. [66] used 150 ml of venous blood followed by centrifugation at 1800 rpm for 15 min and a further centrifugation at 3500 rpm for 10 min, resulting in 20 ml of PRP (four units of 5 ml each). Dallari et al. [63] collected 150 ml of peripheral blood and performed two centrifugations.The first centrifugation to separate erythrocytes from platelets was performed at 1480 rpm for 6 min, the second to concentrate them was performed at 3400 rpm for 15 min. They further added 1 ml of calcium chloride to activate platelets [63]. Doria et al. [64] performed two centrifugations, lasting 6 and 15 min, without adding calcium chloride [64]. Calcium chloride (CaCl_2_) is an exogenous coagulation factors which aims to clot the PRP [67, 68]. However, its use is still debated, and consensus has not been reached. Further investigations to validate and standardise PRP preparation procedures are required. Between-study heterogeneity with regard to the timing of the injection was also detected. Some authors performed PRP injections during meniscal repair [25, 27, 28, 30]. To achieve closer contact between PRP and the meniscal lesion, Dai et al. [25] performed the injections after the meniscal suture but before those sutures were fastened. Kaminski et al. [30] performed PRP injections during the meniscal repair in an arthroscopically guided fashion. Duif et al. [26] performed PRP injections after the arthroscopic procedure with a sterile syringe through the anterolateral portal. In contrast, Kemmochi et al. [20] injected the PRP before the arthroscopic procedure. These differences between protocols may produce marked clinical differences and, given the limited quantitative data available, further subgroup analyses were not possible.

## Conclusion

The current evidence does not support PRP augmentation when performing arthroscopic meniscal repair. At approximately 2 years follow-up, PRP augmentation demonstrated similar VAS, Lysholm, and IKDC scores compared with isolated arthroscopic meniscal repair. Moreover, no differences were detected in failure and revision rates.

## Data Availability

The data underlying this article are available in the article.

## Abbreviations

PRP: Platelet rich plasma
PROMs: Patient-reported outcome measures
PRISMA: Preferred Reporting Items for Systematic Reviews and Meta-Analyses
VAS: Analogue scale
IKDC: International Knee Documentation Committee
MD: Mean difference
OR: Odds ratio
CI: Confidence interval
